# The effect of birth weight and time of day on the thermal response of newborn water buffalo calves

**DOI:** 10.3389/fvets.2023.1084092

**Published:** 2023-02-28

**Authors:** Fabio Napolitano, Andrea Bragaglio, Ada Braghieri, Ayman H. Abd El-Aziz, Cristiane Gonçalves Titto, Dina Villanueva-García, Patricia Mora-Medina, Alfredo M. F. Pereira, Ismael Hernández-Avalos, Nancy José-Pérez, Alejandro Casas-Alvarado, Karina Lezama-García, Adriana Domínguez-Oliva, Daniela Rodríguez-González, Aldo Bertoni, Daniel Mota-Rojas

**Affiliations:** ^1^Scuola di Scienze Agrarie, Forestali, Alimentari ed Ambientali, Università degli Studi della Basilicata, Potenza, Italy; ^2^Consiglio per la Ricerca in Agricoltura e l'Analisi Dell'Economia Agraria (CREA), Research Centre for Engineering and Food Processing, Treviglio, Italy; ^3^Animal Husbandry and Animal Wealth Development Department, Faculty of Veterinary Medicine, Damanhour University, Damanhour, Egypt; ^4^Laboratório de Biometeorologia e Etologia, FZEA-USP, Faculdade de Zootecnia e Engenharia de Alimentos, Universidade de São Paulo, Pirassununga, Brazil; ^5^Division of Neonatology, Hospital Infantil de México Federico Gómez, Mexico City, Mexico; ^6^Facultad de Estudios Superiores Cuautitlán, Universidad Nacional Autónoma de México (UNAM), Cuautitlán, Mexico; ^7^Mediterranean Institute for Agriculture, Environment and Development (MED), Institute for Advanced Studies and Research, Universidade de Évora, Évora, Portugal; ^8^Neurophysiology, Behavior and Animal Welfare Assessment, DPAA, Universidad Autónoma Metropolitana (UAM), Mexico City, Mexico

**Keywords:** body temperature, infrared thermography, newborns, thermoregulation, hypothermia

## Abstract

During the 1st days of life, water buffalo calves, especially those with low birth weight, are susceptible to hypothermic mortality due to scarce energy reserves provided by fats. This means that monitoring the thermal state of newborns is essential. The objectives of the present study were to apply infrared thermography (IRT) in 109 buffalo calves to detect differences in the surface temperatures of six thermal windows –lacrimal gland, lacrimal caruncle, periocular region, nostrils, ear canal, pelvic limbs–, and determine their association to birth weight during the first 6 days of life. The calves were divided into four categories according to their weight (Q_1_, 37.8–41.25 kg; Q_2_, 41.3–46.3 kg; Q_3_, 46.4–56.3 kg; Q_4_, 56.4–60.3 kg). The thermographic images were recorded in the morning and afternoon. Results showed that the animals in Q_4_ registered the highest temperatures in all the thermal windows, and that these were higher in the afternoon (*p* < 0.0001). When considering the thermal windows, those located in the facial region recorded the highest temperatures; in contrast, the temperatures at the pelvic limbs remained below the average values of the other windows (33.41 and 33.76°C in the morning and afternoon, respectively). According to these results, the birth weight of water buffaloes is a factor that alters their thermoregulation during the 1st days of life, a condition that can be partially compensated by colostrum intake to promote development of an efficient thermoregulatory mechanism in water buffalo calves.

## 1. Introduction

At birth, water buffalo calves face extrauterine temperatures that can be 10–15°C below their core temperature (1). During this critical period, maintaining body temperature is essential for survival (2). However, to achieve efficient thermoregulation, animals require energy reserves or energy production that depend on the characteristics of individuals at birth (3) and the modulations of this parameter are closely related to stability of cellular, muscular, nervous endocrine, renal, cardiovascular and respiratory functions (4), and ensured by mechanisms of thermolysis and thermogenesis (5). Not only dams' weight at parturition (6), but also birthweight is a risk factor related to neonatal mortality, especially in neonates (7) born with weights below the average range (38.2 ± 0.2 kg in buffalo calves) (8), as this exposes them to hypothermia due to a high specific surface area and reduced thermogenic capacity (9, 10). In addition, birth weight is associated with the capacity for adequate growth and development of buffaloes throughout their productive life (11). Calves with lower weight may also show a decrease in colostrum consumption compared to animals with medium to high weight. The colostrum is the main source of energy for newborn ruminants (12), and reduced energy resources affect non-shivering thermogenesis, which is the main mechanism for heat production in these neonates (3, 13). Certain morphological characteristics of the water buffalo, i.e., scarce hair, thick epidermis, high melanin concentration, and the small number and large size of sweat glands, all favor the development of thermoregulatory mechanisms that differ from those commonly observed in conventional cattle (*Bos taurus* and *Bos indicus*) (14). Other elements that influence this response are environmental temperatures and the weather, as windy and rainy conditions at the beginning of spring or winter impact the thermoregulation process (15, 16).

Given these effects, evaluating the temperature of newborns offers a way to identify state of hypothermia and prevent the consequences of hypothermia (3). Assessing the surface temperature of animals by means of infrared thermography (IRT) has been shown to be an effective tool to non-invasively determine the peripheral thermoregulatory response of species such as puppies (6), Holstein Friesian calves (17), and piglets (5, 18). However, applications of this technique in water buffaloes are limited, and existing studies have not established the relationship between time of the day, and weight on the thermoregulatory mechanisms of this species. Therefore, the objectives of the present study were: (i) to evaluate, by means of IRT, surface temperatures in six thermal windows of calves from birth to 6 days of life; (ii) to establish the relationship between the birth weight of water buffaloes and their thermoregulatory capacity during the 1st days of life; and (iii) to evaluate the effect of the time of day on changes in the microcirculation of calves.

## 2. Materials and methods

### 2.1. Location

This study was conducted in a buffalo production unit in south-eastern Mexico, at an elevation of 10 m above sea level. The zone is characterized by a subtropical wet climate or Cfa, according to the Koppen-Geiger classification, with an average temperature of 27°C and annual rainfall of 2,900 mm. The experimental periods were September to November 2020 and September to November 2021. The ambient temperature during the evaluation period had average maximum and minimum values of 36°C and 23°C. Maximum and minimum percentages of relative humidity fluctuated from 81 to 94%, respectively.

### 2.2. Study population

To evaluate calves from birth (day 0), 123 dam water buffaloes (*Bubalus bubalis*) of the Buffalypso breed, close to calving, were monitored. One week before calving, the animals were moved to a 5,000 m^2^ maternity paddock (pasture) with a capacity of 20 buffaloes per group. The females were fed forage native to the region *(Paspalum fasciculatum* and *Hymenachne amplexicaulis*) with 50 g of mineral supplement. At birth, the calves were distributed into four groups according to quartiles (18–21). In total, 109 calves were included in this study. Fourteen female buffaloes and their calves were excluded because their agonistic temperament made it impossible to approach the calves to obtain the required measurements.

Immediately after the first intake of colostrum and formation of the mother-calf bond (observed as licking and ingestion of amniotic fluids and placental membranes by the dams) (7), the calves were weighed using a 100-kg Outmate Digital Crane Scale^®^ (Data Weighing Systems, USA) with a readout accuracy of 0.1 lb/0.05 kg-grams. Newborns were then divided into four experimental groups according to birth weight in each quartile. The first group (Q_1_) included 25% of the calves with the lowest values recorded; quartile two (Q_2_) included the lowest 25% of the mean; quartile three (Q_3_) included 25% of values above the mean; and quartile four (Q_4_) included 25% of the highest values. In this way, the distribution in quartiles was: Q_1_ (37.8–41.2 kg, *n* = 25 calves); Q_2_ (41.3–46.3 kg, *n* = 29 calves); Q_3_ (46.4–56.3 kg, *n* = 29 calves); and Q_4_ (56.4–60.3 kg, *n* = 26 calves).

### 2.3. Infrared thermography

**Figure 1:**
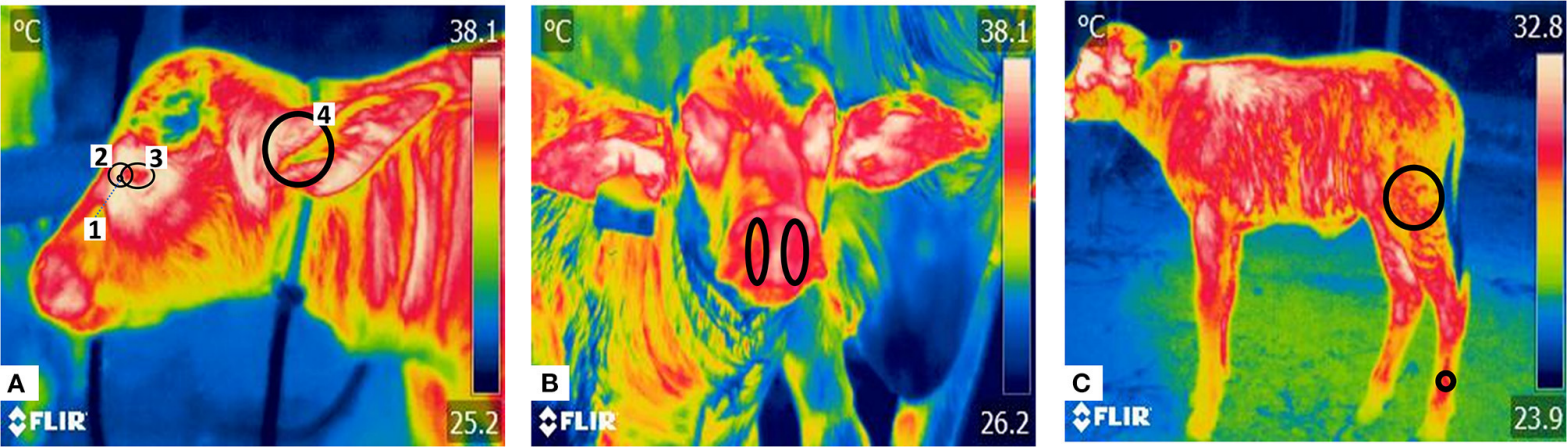
Thermal windows in newborn water buffaloes. Thermal windows captured in three radiometric images (lateral facial, frontal facial, and latero-lateral). **(A)** The lateral facial view shows four thermal windows: (1) the lacrimal gland, delimited by a circle in the medial quadrant of the ocular orbit; (2) the lacrimal caruncle, delimited by a circle around the lacrimal gland, from the medial region of the eye toward the rostral area of the palpebral commissure; (3) the periocular region, outlining the upper and lower eyelids in an ellipse; and (4) the auditory canal, marked with a circle covering the entire acoustic meatus. **(B)** Frontal facial region with delimitation of the nostrils. The temperature irradiated from this area was registered by drawing an ellipse based on each nostril's upper and lower end. **(C)** Latero-lateral region, where two thermal windows can be observed. The pelvic limb is delimited by two circles: one placed at the insertion of the *M. biceps femoris* and the second at the distal end, 10 cm above the hooves, located in the *superficial digital flexor muscle*. The average values of these measures were obtained.

Radiometric images were taken with a thermal camera model FLIR^®^ Thermal TM E80 (FLIR Systems, USA), with a resolution of 320 × 240 pixels, thermal sensitivity of <0.045°C, precision ± 2°C or 2% and an emissivity of 0.95. Each radiometric capture was performed at 1–2 m from the calf, focusing on three regions: lateral facial, frontal facial, and left or right latero- lateral. Six thermal windows were assessed: lacrimal gland, lacrimal caruncle, periocular region, nostrils, ear canal, and pelvic limbs (Figure 1). The measurement of surface temperatures in these windows was carried out for a period of 6 days, from the day of birth (day 0) to the 5th day postpartum (day 5).

Thermographic monitoring was performed twice: from 8:00 a.m. to 9:00 a.m. and from 4:00 p.m. to 5:00 p.m. in the central zone of Mexico. The ambient temperature during the September-November period ranged from 23 to 27°C in the morning (8:00-−9:00 a.m.) to 32–36°C (4:00 to 5:00 p.m.) in the afternoon. Relative humidity fluctuated from 81 to 93% in the morning to 82–94% in the afternoon. During the thermographic monitoring, neonates and their dams remained in the maternity paddocks all the time. The thermal imaging sequence was performed in the paddock. Buffalo calves were not moved or handled from their birthplace, so the thermal evaluation was carried out directly on the grass. Thermal images were taken in the following sequence: the camera placed in front of the animal to take the frontal facial region, then to the side to obtain the lateral facial view. Subsequently, the left latero-lateral and the right latero-lateral regions were captured on each side of the animal.

All radiometric images were stored in JPG format for later analysis using FLIR Tools Systems, USA^®^ software to set points in the proposed thermal windows to obtain maximum, minimum, and average temperatures from each calf on each sampling day during the mornings and afternoons.

### 2.4. Statistical analysis

The GraphPad Prism statistical package (ver. 9.4.0) was used to obtain descriptive statistics for the weight of the animals (Q_1_, Q_2_, Q_3_, Q_4_), time of day (morning, afternoon), thermal window, and age of the animals (days 0, 1, 2, 3, 4, 5). The Shapiro-Wilk test was performed to analyze the normality of the data. The weight and age of the calves and the time of day were considered as independent variables, whereas the temperature of the thermal windows was considered a dependent variable. To evaluate the effects of these variables, analysis of variance (ANOVA) was used in a mixed linear model:


$$\begin{array}{l} Y_{\text{ijkl}}=\mu +W_{\text{i}}+T_{\text{j}}+A_{\text{k}}+W.T.\text{Aijk}+\beta \text{l}+e_{\text{ijk}} \end{array}$$

where:

Y = response variable (temperature of the thermal windows).

W_i_ = effect of the weight of the animals (Q_1_, Q_2_, Q_3_, Q_4_).

T_j_ = effect of time of day (morning, afternoon).

A_k_= effect of the animals' age (days 0, 1, 2, 3, 4, 5).

β= random effect (per animal).

μ = population mean.

e = residue.

Correlations between the time of day and thermal window temperatures on day 0 were obtained using Spearman correlation. Tukey's *post-hoc* test was adopted to evaluate the differences between means. In all significance level of *p* < 0.05 was set.

### 2.5. Ethics statement

The animals monitored in this study were not touched or stressed, as infrared thermography is a non-invasive technique; therefore, the protocol did not require approval from an Ethics Committee. During the study, handling of the animals was carried out under the guidelines of the Official Mexican Standard, NOM-062-ZOO-1999, which stipulates the technical specifications for the production, care, and ethical use of animals.

## 3. Results

A total of 23,544 recordings of maximum, minimum, and average surface temperatures were obtained from newborn water buffaloes during the first 6 days of life. In general, significant differences were observed among the six thermal windows due to the weight and time of day. These differences are described as follows.

### 3.1. Effect of weight on dermic microcirculation changes in newborn buffaloes during the 1st days of life

Table 1 lists the thermal responses of the pelvic limb region. On the morning of day 0, the surface temperature was 1.49°C higher in Q_3_ and Q_4_ than in Q_1_ and Q_2_ (*p* < 0.05). A similar pattern was observed on the following days, were the lowest temperatures occurred on day 0 but increased by 4.53°C in Q_1_ and 6.43°C in Q_4_ on day 1. On the afternoon of day 0, the lowest temperatures were observed on this day, but they showed significant increases (*p* < 0.05) of 4.35°C and 6.43 °C in Q_1_ and Q_4_, respectively, on day 1. The surface temperatures of the pelvic limb in Q_2_ were 0.74, 0.94, and 1.19°C lower than those in Q_1_, Q_3_, and Q_4_, respectively (*p*< 0.05). This response was observed in the following days since heavier calves (Q_3_ and Q_4_) maintained higher temperatures until the end of the evaluation period.

**Table 1:** Mean pelvic limb surface temperature ± standard error (SE) in newborn water buffaloes (*n* = 109) at different birth weights and daytimes (morning/afternoon) during the first 6 days of life.

**Day**	**Q**_**1**_		**Q**_**2**_		**Q**_**3**_		**Q**_**4**_	
	***n*** = **25**		***n** =* **29**		***n** =* **29**		***n** =* **26**	
	Morning	Afternoon	Morning	Afternoon	Morning	Afternoon	Morning	Afternoon
0	28.97 ± 0.07^3, a*^	29.62 ± 0.07^3, a^	28.44 ± 0.08^3, b*^	28.88 ± 0.08^3, b*^	29.62 ± 0.06^3, c*^	29.82 ± 0.06^3, a^	29.93 ± 0.08^2, d, *^	30.07 ± 0.08^3, a^
1	33.32 ± 0.11^1, c*^	33.97 ± 0.11^1, c^	33.13 ± 0.13^1, c*^	33.57 ± 0.14^1, 2, c*^	35.96 ± 0.09^1, b*^	36.16 ± 0.09^1, b^	36.36 ± 0.07^1, a*^	36.50 ± 0.07^1, a^
2	33.44 ± 0.14^1, b*^	34.10 ± 0.14^1, b^	32.90 ± 0.14^1, c*^	33.34 ± 0.15^1, c*^	35.38 ± 0.19^1, 2, a^	35.58 ± 0.19^1, 2, a^	35.88 ± 0.11^1, a*^	36.03 ± 0.11^1, 2, a^
3	32.20 ± 0.17^2, b*^	32.86 ± 0.17^2, b^	31.91 ± 0.14^2, b*^	32.34 ± 0.14^2, b^	35.05 ± 0.13^2, a*^	35.25 ± 0.13^2, a^	35.40 ± 0.07^1, a*^	35.55 ± 0.07^2, a^
4	33.35 ± 0.11^1, b^	34.00 ± 0.11^1, b^	32.93 ± 0.11^1, b^	33.37 ± 0.11^1, c^	34.93 ± 0.15^2, a^	34.93 ± 0.15^2, a^	35.13 ± 0.15^1, a^	35.38 ± 0.13^2, a^
5	33.23 ± 0.13^1, b*^	33.88 ± 0.13^1, b^	32.69 ± 0.13^1, 2, b*^	33.13 ± 0.13^1, 2, c^	35.46 ± 0.17^1, 2, a*^	35.66 ± 0.17^1, a^	35.94 ± 0.14^1, a*^	36.08 ± 0.15^1, 2, a^

Linear mixed model analysis with a Tuckey *post-hoc* test using the GraphPad program (VER. 9.4.0).

n, number of calves; Weight of calves according to category: Q_1_, 37.8–41.2 kg; Q_2_, 41.3–46.3 kg; Q_3_, 46.4–56.3 kg; Q_4_, 56.4–60.3 kg. Mean ± standard error.

a, b, c, d Different lowercase indicate a statistical difference between groups.

1, 2, 3 Different numbers indicate statistically significant differences between days in the same weight group.

* Indicates a significant statistical difference in temperature due to the effect of time of day.

Regarding the surface temperature of the lacrimal caruncle (Table 2), in the morning, significant differences (*p* < 0.05) were observed among the quartiles and over time. On the morning of day 0, Q_4_ was found to be 0.9°C higher than Q_2_ (*p* < 0.05), and it can be observed that Q_4_ did not show significant variations in the mornings of the days evaluated. On day 1, 2, and 3, buffalo calves from Q_3_ and Q_4_ showed the highest temperatures when compared to Q_1_ and Q_2_ (*p* < 0.05). On days 4 and 5, the temperatures of Q_1_, Q_3_, and Q_4_ were significantly higher than those of Q_2_ (*p* < 0.05). Readings from the afternoon of day 1 showed that Q_1_, Q_3_, and Q_4_ presented a temperature 0.7°C higher than those of Q_2_ (*p* < 0.05). Interestingly, at day 5, calves from Q_2_ had lower lacrimal caruncle temperature than the rest of the groups, although a periodically increasing was reported during the 6 days (*p* < 0.05).

**Table 2:** Mean lacrimal caruncle surface temperature ± standard error (SE) in 109 newborn water buffaloes at different birth weights and daytimes (morning/afternoon) during the first 6 days of life.

**Day**	**Q**_**1**_		**Q**_**2**_		**Q**_**3**_		**Q**_**4**_	
	***n*** = **25**		***n** =* **29**		***n** =* **29**		***n** =* **26**	
	Morning	Afternoon	Morning	Afternoon	Morning	Afternoon	Morning	Afternoon
0	36.75 ± 0.12^1, 2, a, *^	37.40 ± 0.12^1, 2, a^	36.23 ± 0.11^1, 2, b*^	36.67 ± 0.12^1, 2, a^	37.0 ± 0.09^1, 2, a, b*^	37.20 ± 0.09^1, a^	37.17 ± 0.06^1, a*^	37.31 ± 0.06^1, a^
1	36.49 ± 0.05^2, a*^	37.14 ± 0.05^2, a^	35.99 ± 0.09^2, a*^	36.42 ± 0.09^2, a^	36.96 ± 0.06^2, a*^	37.16 ± 0.06^1, a^	37.09 ± 0.08^1, a*^	37.23 ± 0.08^1, a^
2	36.72 ± 0.18^1, 2, a*^	37.38 ± 0.18^1, 2, a, b^	36.18 ± 0.17^1, 2, a*^	36.62 ± 0.18^1, 2, a, b^	37.02 ± 0.12^1, a*^	37.22 ± 0.12^1, a^	37.38 ± 0.09^1, a*^	37.52 ± 0.09^1, a, b^
3	36.23 ± 0.10^2, a*^	36.88 ± 0.11^2, a, b^	35.87 ± 0.09^2, a*^	36.31 ± 0.10^2, a^	37.14 ± 0.06^1, a*^	37.34 ± 0.06^1, a^	37.36 ± 0.06^1, a*^	37.50 ± 0.06^1, a^
4	36.78 ± 0.11^1, a, b*^	37.43 ± 0.11^1, 2, a^	36.27 ± 0.11^1, 2, b*^	36.71 ± 0.11^1, 2, a^	37.04 ± 0.06^1, 2, a*^	37.24 ± 0.06^1, a, b^	37.26 ± 0.05^1, a*^	37.40 ± 0.05^1, a, b^
5	36.94 ± 0.06^1, a*^	37.60 ± 0.06^1, a^	36.53 ± 0.08^1, a*^	36.97 ± 0.09^1, a^	37.02 ± 0.04^1, 2, a*^	37.22 ± 0.04^1, a, b^	37.28 ± 0.04^1, a*^	37.43 ± 0.03^1, a, b^

Linear mixed model analysis with a Tuckey *post-hoc* test using the GraphPad program (VER. 9.4.0).

n, number of calves; Weight of calves according to category: Q_1_, 37.8–41.2 kg; Q_2_, 41.3–46.3 kg; Q_3_, 46.4–56.3 kg; Q_4_, 56.4–60.3 kg. Mean ± standard error.

a, b Different lowercase indicate a statistical difference between groups.

1, 2 Different numbers indicate statistically significant differences between days in the same weight group.

* Indicates a significant statistical difference in temperature due to the effect of time of day.

The surface temperature readings from the periocular region showed differences among quartiles and over time (Table 3). Regarding the difference among the four weight groups, in the morning of day 0, temperatures in Q_3_ and Q_4_ were 1.6°C higher than in Q_1_ and Q_2_ (*p* < 0.05). Although a progressive increase in temperature was recorded in all groups [e.g., the temperature of Q_1_ on the morning of day 5 increased 0.6°C compared to day 3 (*p* < 0.05)], on day 5, Q_1_, Q_3_, and Q_4_ were 0.7°C higher than Q_2_ (*p* < 0.05). This response was also observed during the afternoon of day 5, when Q_1_ and Q_4_ were 0.6°C higher than Q_2_ (*p* < 0.05) while Q_3_ and Q_4_ recorded the highest values during all the evaluation days (*p* < 0.05). It is important to mention here that in contrast to Q_1_, Q_4_ only showed a decrease of 0.91°C in the morning and afternoon of day 1 but then remained without any significant variation until the end of the evaluation period.

**Table 3:** Mean periocular surface temperature ± standard error (SE) in 109 newborn water buffaloes at different birth weights and daytimes (morning/afternoon) during the first 6 days of life.

**Day**	**Q**_**1**_		**Q**_**2**_		**Q**_**3**_		**Q**_**4**_	
	***n*** = **25**		***n** =* **29**		***n** =* **29**		***n** =* **26**	
	Morning	Afternoon	Morning	Afternoon	Morning	Afternoon	Morning	Afternoon
0	35.63 ± 0.06^1, 2, b*^	36.28 ± 0.06^1, 2, b^	35.26 ± 0.07^1, b*^	35.70 ± 0.07^1, c^	37.01 ± 0.11^1, a*^	37.21 ± 0.11^1, a^	37.21 ± 0.07^1, a*^	37.35 ± 0.08^1, a^
1	35.66 ± 0.04^1, 2, b*^	36.31 ± 0.04^1, 2, a^	35.20 ± 0.06^1, c*^	35.64 ± 0.06^1, b^	36.10 ± 0.03^2, a*^	36.30 ± 0.03^2, a^	36.30 ± 0.04^2, a*^	36.44 ± 0.04^2, a^
2	35.90 ± 0.16^1, 2, a, b*^	36.55 ± 0.16^1, 2, a^	35.29 ± 0.16^1, b*^	35.73 ± 0.17^1, b^	36.13 ± 0.10^2, a*^	36.32 ± 0.10^2, a, b^	36.30 ± 0.08^2, a*^	36.44 ± 0.08^2, a^
3	35.40 ± 0.09^2, b*^	36.06 ± 0.10^2, b, c^	35.12 ± 0.10^1, b*^	35.56 ± 0.10^1, c^	36.38 ± 0.08^2, a*^	36.58 ± 0.08^2, a, b^	36.52 ± 0.06^2, a*^	36.66 ± 0.07^2, a^
4	35.90 ± 0.07^1, 2, a, b*^	36.55 ± 0.07^1, 2, a, b^	35.47 ± 0.08^1, b*^	35.90 ± 0.09^1, b^	36.35 ± 0.10^2, a*^	36.55 ± 0.10^2, a^	36.40 ± 0.10^2, a*^	36.54 ± 0.10^2, a, b^
5	36.06 ± 0.10^1, a, b*^	36.71 ± 0.10^1, a^	35.54 ± 0.11^1, b*^	35.98 ± 0.11^1, b^	36.22 ± 0.08^2, a*^	36.42 ± 0.08^2, a, b^	36.42 ± 0.05^2, a*^	36.56 ± 0.05^2, a^

Linear mixed model analysis with a Tuckey *post-hoc* test using the GraphPad program (VER. 9.4.0).

n, number of calves; Weight of calves according to category: Q_1_, 37.8–41.2 kg; Q_2_, 41.3–46.3 kg; Q_3_, 46.4–56.3 kg; Q_4_, 56.4–60.3 kg. Mean ± standard error.

a, b Different lowercase indicate a statistical difference between groups.

1, 2 Different numbers indicate statistically significant differences between days in the same weight group.

* Indicates a significant statistical difference in temperature due to the effect of time of day.

Regarding the thermal response of the auditory canal in the water buffalo calves (Table 4), during the morning of the six evaluation days Q_3_ and Q_4_ had higher temperature than Q_1_ and Q_2_, (*p* < 0.05). The same response was observed in the afternoon, and only on day 5, the temperatures of Q_1_ and Q_4_ were 0.3°C higher than those obtained in Q_2_ and Q_3_ (*p* < 0.05). The series of readings taken over time indicated that Q_1_ did not present significant differences until day 2, when an increase of 1.91°C was found. In contrast, Q_4_ showed a significant decrease of 1.65°C. on day 1, followed by a significant increase of 0.96°C on day 2. Q_1_ and Q_4_ only presented significant variations toward the end of the afternoon evaluations, but not during the morning study period, when Q_1_ tended to show a decrease on day three, with a difference of 1.06°C with respect to day 2. Later, the temperature began to increase, by 0.94 and 0.99°C on days 4 and 5, respectively.

**Table 4:** Mean auditory canal surface temperature ± standard error (SE) in 109 newborn water buffaloes at different birth weights and daytimes (morning/afternoon) during the first 6 days of life.

**Day**	**Q**_**1**_		**Q**_**2**_		**Q**_**3**_		**Q**_**4**_	
	***n*** = **25**		***n** =* **29**		***n** =* **29**		***n** =* **26**	
	Morning	Afternoon	Morning	Afternoon	Morning	Afternoon	Morning	Afternoon
0	34.03 ± 0.19^2, 3, b*^	34.68 ± 0.19^2, 3, b^	33.62 ± 0.21^2, 3, b*^	34.06 ± 0.21^2, 3, b^	36.58 ± 0.22^1, 2, a*^	36.78 ± 0.22^1, 2, a^	37.07 ± 0.07^1, a*^	37.21 ± 0.07^1, a^
1	33.43 ± 0.05^3, b*^	34.08 ± 0.06^3, b^	32.96 ± 0.09^3, c*^	33.39 ± 0.10^3, b^	35.29 ± 0.11^4, a*^	35.49 ± 0.11^4, a^	35.42 ± 0.13^4, a*^	35.56 ± 0.14^4, a^
2	35.34 ± 0.34^1, a, b*^	35.99 ± 0.34^1, 2, a, b^	34.45 ± 0.36^1, 2, b*^	34.89 ± 0.36^1, 2, b^	35.93 ± 0.20^3, a, b*^	36.13 ± 0.20^3, a, b^	36.38 ± 0.10^2, 3, a*^	36.52 ± 0.09^2, 3, a^
3	34.28 ± 0.27^2, 3, b*^	34.94 ± 0.27^2, 3, b^	34.13 ± 0.23^2, b*^	34.57 ± 0.22^2, b^	36.61 ± 0.06^1, a*^	36.81 ± 0.06^1, a^	36.79 ± 0.05^1, 2, a*^	36.93 ± 0.05^1, 2, a^
4	35.22 ± 0.26^1, 2, b*^	35.88 ± 0.26^1, 2, a, b^	34.92 ± 0.21^1, 2, b*^	35.36 ± 0.22^1, 2, b^	36.29 ± 0.06^2, 3, a*^	36.49 ± 0.06^2, 3, a^	36.49 ± 0.06^2, 3, a*^	36.63 ± 0.05^2, 3, a^
5	36.21 ± 0.09^1, a*^	36.86 ± 0.09^1, a^	35.68 ± 0.11^1, b*^	36.12 ± 0.11^1, b^	36.26 ± 0.06^2, 3, a^	36.46 ± 0.06^2, 3, b*^	36.37 ± 0.06^3, a*^	36.51 ± 0.07^3, a^

Linear mixed model analysis with a Tuckey *post-hoc* test using the GraphPad program (VER. 9.4.0).

n, number of calves; Weight of calves according to category: Q_1_, 37.8–41.2 kg; Q_2_, 41.3–46.3 kg; Q_3_, 46.4–56.3 kg; Q_4_, 56.4–60.3 kg. Mean ± standard error.

a, b Different lowercase indicate a statistical difference between groups.

1, 2, 3 Different numbers indicate statistically significant differences between days in the same weight group.

* Indicates a significant statistical difference in temperature due to the effect of time of day.

The surface temperature in the nostrils, as shown in Table 5, had the lowest values during the morning of day 0 in Q_1_ and Q_2_ (*p* < 0.05). Consecutively, on day 5, Q_3_ and Q_4_ were 1.4°C higher than Q_1_ and Q_2_. When considering afternoon temperatures, they had a similar pattern between groups. On day 0, the temperatures of Q_3_ and Q_4_ were 0.8°C higher than Q_1_ and 1.4°C higher than Q_2_. This response was maintained during the rest of the days, and when comparing events, observations showed that Q_1_, Q_3_, and Q_4_ had significantly higher morning and afternoon temperatures on day 0 than on day 1 (*p* < 0.05). The temperature on day 1 was between 0.9 and 1°C higher than on days 2, 3, 4, and 5 (*p* < 0.05). Regarding Q_2_, readings showed that in both the morning and afternoon of day 0 the temperatures were 1.1°C higher than on day 1 (*p* < 0.05), and that on the same day there was a difference of 1°C compared to day 3 (*p* < 0.05).

**Table 5:** Mean nostrils surface temperature ± standard error (SE) in 109 newborn water buffaloes at different birth weights and daytimes (morning/afternoon) during the first 6 days of life.

**Day**	**Q**_**1**_		**Q**_**2**_		**Q**_**3**_		**Q**_**4**_	
	***n*** = **25**		***n** =* **29**		***n** =* **29**		***n** =* **26**	
	Morning	Afternoon	Morning	Afternoon	Morning	Afternoon	Morning	Afternoon
0	35.33 ± 0.07^1, b*^	35.98 ± 0.07^1, b^	34.89 ± 0.06^1, c*^	35.32 ± 0.06^1, c^	36.57 ± 0.07^1, a*^	36.77 ± 0.07^1, a^	36.75 ± 0.07^1, a*^	36.89 ± 0.08^1, a^
1	34.35 ± 0.12^2, c*^	35.00 ± 0.12^2, b^	33.79 ± 0.11^2, c*^	34.23 ± 0.11^2, c^	35.88 ± 0.11^2, b*^	36.08 ± 0.11^2, a^	36.24 ± 0.05^2, a*^	36.38 ± 0.05^2, a^
2	33.54 ± 0.11^3, b*^	34.19 ± 0.11^3, b^	32.90 ± 0.23^2, 3, b*^	33.33 ± 0.23^2, 3, b^	34.56 ± 0.19^3, a*^	34.76^3, a^ ± 0.19	35.06 ± 0.13^3, a*^	35.20 ± 0.14^3, a^
3	33.24 ± 0.12^3, b*^	33.90 ± 0.13^3, b^	32.77 ± 0.13^3, b*^	33.21 ± 0.13^3, c^	34.27 ± 0.12^3, a*^	34.47 ± 0.12^3, a, b^	34.65 ± 0.06^3, 4, a*^	34.79 ± 0.07^3, 4, a^
4	33.58 ± 0.12^3, b, c*^	34.23 ± 0.12^3, a^	33.27 ± 0.12^2, 3, c*^	33.70 ± 0.13^2, 3, a^	34.26 ± 0.12^3, a, b*^	34.46 ± 0.12^3, a^	34.54 ± 0.11^4, a*^	34.68 ± 0.11^4, a^
5	33.30 ± 0.12^3, b*^	33.95 ± 0.12^3, b^	33.02 ± 0.12^2, 3, b*^	33.46 ± 0.12^2, 3, b^	34.27 ± 0.11^3, a*^	34.47 ± 0.11^3, a^	34.74 ± 0.08^3, 4, a*^	34.88 ± 0.08^3, 4, a^

Linear mixed model analysis with a Tuckey *post-hoc* test using the GraphPad program (VER. 9.4.0).

n, number of calves; Weight of calves according to category: Q_1_, 37.8–41.2 kg; Q_2_, 41.3–46.3 kg; Q_3_, 46.4–56.3 kg; Q_4_, 56.4–60.3 kg. Mean ± standard error.

a, b, c Different lowercase indicate a statistical difference between groups.

1, 2, 3 Different numbers indicate statistically significant differences between days in the same weight group.

* Indicates a significant statistical difference in temperature due to the effect of time of day.

The lacrimal gland showed significant differences on the morning of day 0, where Q_3_ and Q_4_ were 0.7°C higher than Q_1_ and 0.2°C higher than Q_2_, respectively (*p* < 0.05; Table 6). During the rest of the days, temperatures of calves belonging to Q_3_ and Q_4_ were higher than Q_1_ and Q_2_ (*p* < 0.05). On the afternoon of days 0 and 5, the temperatures of Q_1_, Q_3_, and Q_4_ were significantly higher than those in Q_2_ (*p* < 0.05), while on day 2, Q_3_ and Q_4_ had temperatures 0.7°C higher than those of Q_1_ and Q_2_ (*p* < 0.05).

**Table 6:** Mean lacrimal gland surface temperature ± standard error (SE) in 109 newborn water buffaloes at different birth weights and daytimes (morning/afternoon) during the first 6 days of life.

**Day**	**Q**_**1**_		**Q**_**2**_		**Q**_**3**_		**Q**_**4**_	
	***n*** = **25**		***n** =* **29**		***n** =* **29**		***n** =* **26**	
	Morning	Afternoon	Morning	Afternoon	Morning	Afternoon	Morning	Afternoon
0	36.86 ± 0.09^1, b*^	37.51 ± 0.09^1, a^	36.33 ± 0.10^1, 2, c*^	36.77 ± 0.10^1, 2, b^	37.28 ± 0.08^1, a*^	37.48 ± 0.08^1, a^	37.40 ± 0.07^1, a*^	37.54 ± 0.07^1, a^
1	36.05 ± 0.09^2, b*^	36.70 ± 0.09^2, b^	35.62 ± 0.11^3, b*^	36.06 ± 0.12^3, c^	37.17 ± 0.07^1, a*^	37.37 ± 0.07^1, a^	37.22 ± 0.10^1, a*^	37.35 ± 0.10^1, a, b^
2	36.50 ± 0.20^1, 2, b*^	37.15 ± 0.20^1, 2, b^	35.96 ± 0.21^1, 2, 3, b*^	36.40 ± 0.22^2, 3, b^	37.13 ± 0.12^1, a*^	37.33 ± 0.12^1, a^	37.34 ± 0.09^1, a*^	37.48 ± 0.08^1, a^
3	35.84 ± 0.22^3, b*^	36.50 ± 0.22^2, b, c^	35.83 ± 0.17^2, 3, b*^	36.27 ± 0.17^2, 3, c^	37.25 ± 0.07^1, a*^	37.45 ± 0.07^1, a, b^	37.47 ± 0.04^1, a*^,	37.62 ± 0.05^1, a^
4	36.77 ± 0.10^1, b, c*^	37.42 ± 0.10^1, a, b^	36.42 ± 0.10^1, 2, c*^	36.86 ± 0.10^1, 2, b^	37.29 ± 0.05^1, a, b*^	37.49 ± 0.05^1, a^	37.42 ± 0.05^1, a*^	37.56 ± 0.05^1, a^
5	36.92 ± 0.07^1, b, c*^	37.57 ± 0.07^1, a^	36.60 ± 0.08^1, c*^	37.03 ± 0.08^1, b^	37.30 ± 0.05^1, a, b*^	37.50 ± 0.05^1, a^	37.40 ± 0.05^1, a*^	37.54 ± 0.05^1, a^

Linear mixed model analysis with a Tuckey *post-hoc* test using the GraphPad program (VER. 9.4.0).

n, number of calves; Weight of calves according to category: Q_1_, 37.8–41.2 kg; Q_2_, 41.3–46.3 kg; Q_3_, 46.4–56.3 kg; Q_4_, 56.4–60.3 kg. Mean ± standard error.

a, b, c, d Different lowercase indicate a statistical difference between groups.

1, 2, 3 Different numbers indicate statistically significant differences between days in the same weight group.

* Indicates a significant statistical difference in temperature due to the effect of time of day.

### 3.2. Thermal variation according to body region

In all groups, the lacrimal gland and lacrimal caruncle had the highest temperatures. In Q_2_, the lacrimal caruncle had significantly higher temperatures, with a difference of 0.16°C compared to the lacrimal gland. Similarly, the Q_3_ and Q_4_ newborns had temperature differences of 0.2–0.11°C between these two areas (Table 7). With respect to the periocular region, all the study animals had high temperatures in the thermal window, except those in Q_4_, where no significant differences were found with the auditory canal (36.601 ± 0.02 vs. 36.491 ± 0.03). The windows with the lowest temperatures were the nostrils and the pelvic limbs. In these zones, the buffaloes in Q_1_ and Q_2_ showed differences of up to 1.47°C.

**Table 7:** Mean surface temperature of the different thermal windows ± standard error (SE) in water buffaloes (*n* = 109) with different birth weights (Q_1_, 37.8–41.2 kg; Q_2_, 41.3–46.3 kg; Q_3_, 46.4–56.3 kg; Q_4_, 56.4–60.3 kg.).

	**Lacrimal gland**	**Lacrimal caruncle**	**Periocular region**	**Auditory canal**	**Nostrils**	**Pelvic limb**
Q_1n = 25_	36.82 ± 0.05^2, b^	36.98 ± 0.04^2^,ª	36.08 ± 0.03^2, c^	35.08 ± 0.08^3, d^	34.21 ± 0.05^3, e^	32.74 ± 0.10^2, f^
Q_2n = 29_	36.34 ± 0.04^2^,ª	36.40 ± 0.03^3^,ª	35.53 ± 0.03^3, b^	34.51 ± 0.08^4, c^	33.66 ± 0.05^4, d^	32.22 ± 0.09^3, f^
Q_3n = 29_	37.33 ± 0.02^1^,ª	37.13 ± 0.02^2, b^	36.46 ± 0.03^1, c^	36.26 ± 0.04^2, d^	35.07 ± 0.06^2, e^	34.50 ± 0.12^1, e^
Q_4n = 26_	37.44 ± 0.02^1^,ª	37.33 ± 0.02^1, b^	36.60 ± 0.02^1, c^	36.49 ± 0.03^1, c^	35.40 ± 0.05^1, d^	34.89 ± 0.12^1, d^

Linear mixed model analysis with a Tuckey *post-hoc* test using the GraphPad program (VER. 9.4.0).

n, number of calves; Weight of calves according to category: Q_1_, 37.8–41.2 kg; Q_2_, 41.3–46.3 kg; Q_3_, 46.4–56.3 kg; Q_4_, 56.4–60.3 kg. Mean ± standard error.

a, b, c, d, e, f Different lowercase indicate a statistical difference between groups.

1, 2, 3 Different numbers indicate statistically significant differences between days in the same weight group.

Regarding the comparison among weights, the lacrimal gland in Q_1_ had the lowest temperature, with a difference of up to 0.62°C compared to Q_4_. The lacrimal caruncle in Q_1_ and Q_2_ did not show significant differences within the groups, while there were differences in Q_3_ and Q_4_, who had the highest temperatures (37.132 ± 0.02 and 37.331 ± 0.02, respectively). The periocular region, auditory canal, nostrils, and pelvic limbs in Q_2_ had the lowest temperatures, with significant differences of 1.07, 1.95, 1.74, and 2.67°C, respectively, compared to Q_4_.

### 3.3. Effect of the time of day on calf thermoregulation during the 1st days of life

**Figure 2:**
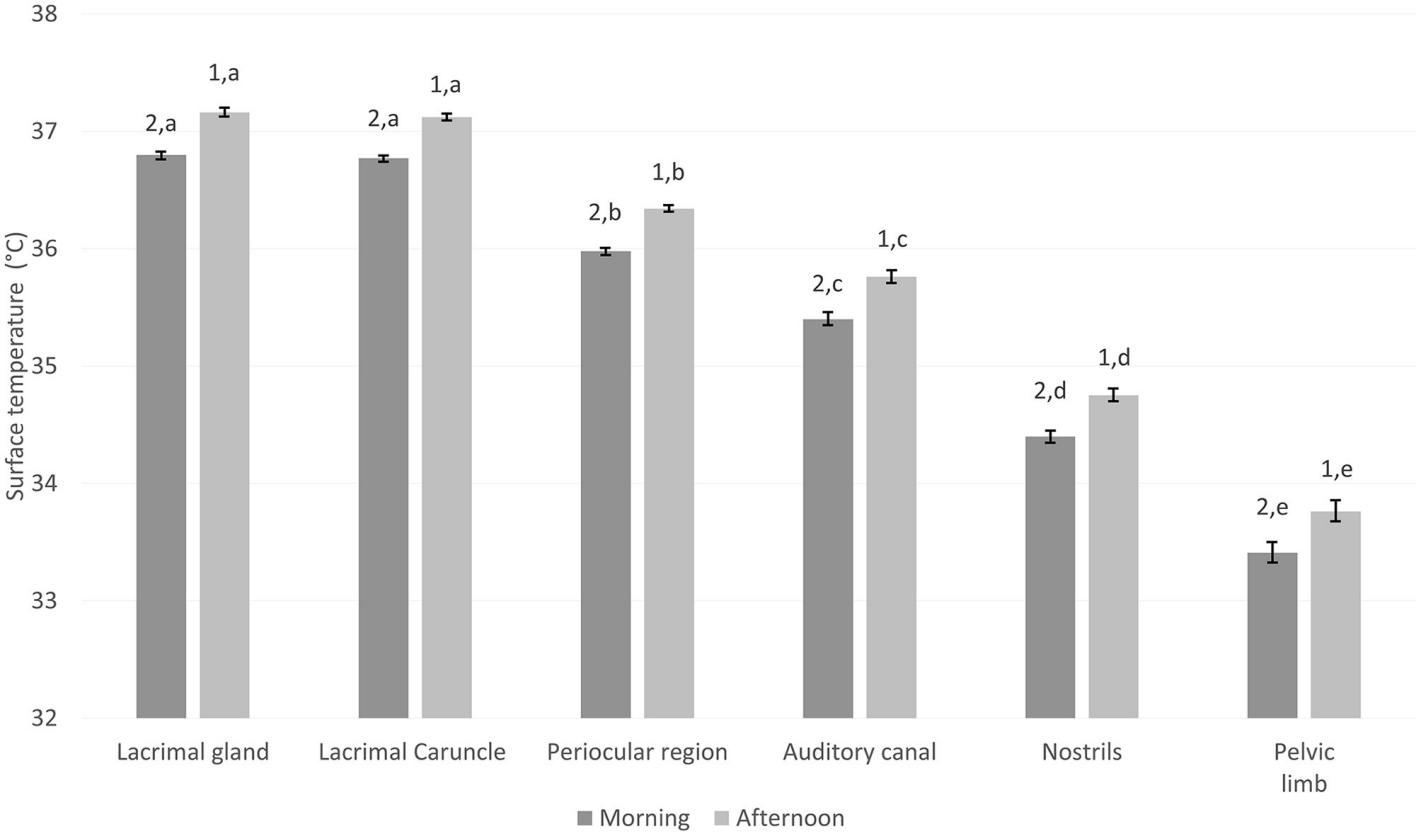
Mean surface temperature ± standard error (SE) of six thermal windows of water buffalo neonates (*n* = 109) at two different times of day (morning from 8:00 to 9:00 a.m., afternoon from 15:00 to 16:00 p.m.). Linear mixed model analysis with a Tuckey *post-hoc* test using the GraphPad program (VER. 9.4.0). *n*, number of calves; Weight of calves according to category: Q_1_, 37.8–41.25 kg; Q_2_, 41.3–46.3 kg; Q_3_, 46.4–56.3 kg; Q_4_, 56.4–60.3 kg. Mean ± standard error. A significance level of *p* < 0.05 was established. ^a,b,c,d,e^Different literals indicate a statistical difference between the weights of the animals. ^1,2^Different numbers indicate statistically significant differences between days in the same weight group.

The temperatures of the lacrimal gland, lacrimal caruncle, periocular region, auditory canal, nostrils, and pelvic limb were 0.3°C higher in the afternoon than the morning (*p* < 0.05; Figure 2). The lacrimal gland and lacrimal caruncle presented significantly higher values than the periocular region, auditory canal, nostrils, and pelvic limb, in both the morning and afternoon (*p* < 0.05). Similarly, the temperatures of the periocular region, auditory canal, nostrils, and pelvic limb differed (*p* < 0.05). Notably, the lowest temperatures were recorded in the pelvic limb (Figure 2).

There was a positive correlation of 0.99 between the surface temperatures of these thermal windows and the time of day (*p* < 0.0001; Table 8).

**Table 8:** Significant correlations between the time of day and the surface temperature of the different thermal windows.

**Thermal window**	**Variable**	**Correlation coefficient (*r*)**	***P*-value**
Periocular	Morning	0.9990	< 0.0001
	Afternoon	0.9988	< 0.0001
Lacrimal gland	Morning	0.9983	< 0.0001
	Afternoon	0.9979	< 0.0001
Lacrimal caruncle	Morning	0.9976	< 0.0001
	Afternoon	0.9976	< 0.0001
Auditory canal	Morning	0.9989	< 0.0001
	Afternoon	0.9984	< 0.0001
Nostrils	Morning	0.9988	< 0.0001
	Afternoon	0.9989	< 0.0001
Pelvic limb	Morning	0.9989	< 0.0001
	Afternoon	0.9974	< 0.0001

Spearman's rank correlation coefficients and their statistical significance between the sampling (morning/afternoon) and the surface temperature of the different thermal windows on day 0, significance level p < 0.

## 4. Discussion

### 4.1. Effect of weight on the thermoregulation of buffalo calves

In this study, the heavier animals in Q_3_ and Q_4_–weight >46 kg—maintained higher surface temperatures than the lighter ones in Q_1_ and Q_2_ (*p* < 0.05). These results show a relationship between birth weight and the thermoregulation of these calves (1), a finding that has been reported for other species, such as pigs and sheep (22, 23). This could be due to a possible difference in the disposition of energy resources, such as brown adipose tissue and glucose, between a low birth weight or a high birth weight animal. In other words, when a newborn has a low birth weight, its response to compensate for hypothermia will be more limited compared to an animal with a higher weight.

Due to the exposure to cold in mammals, a coordinated response is exerted with the Central Nervous System that produces the activation of the sympathetic nervous system, which promotes the neurosecretion of catecholamines (24). Consequently, a response at the vasomotor level is observed with peripheral vasoconstriction to reduce heat loss. In addition to this, a metabolic response is generated due to the interaction with norepinephrine that produces the breakdown of triglycerides in adipocytes that leads to intracellular activation of cAMP and protein kinase A, for the mitochondrial combustion of substrates and the production of heat (25). The concept of thermoneutrality considers the range where the basal metabolism maintains body temperature (26). In this way, the previous explanation would confirm the fact that an animal with a low birth weight would present low levels of substrates to compensate for heat loss and reach the thermal comfort zone.

The association between basal metabolism and the ability to thermoregulate could explain why a low-birth-weight animal has a lower body temperature. In this sense, Diesch et al. (27) showed that calves born in windy and humid regions with an ambient temperature of 10°C delayed the time for first standing. This could be due to the organism using the energetic reserves to activate thermoregulatory mechanisms, such as vasomotor changes, and shivering and non-shivering thermogenesis (1). These mechanisms depend on the animal's metabolic rate, which is lower per unit area in low-weight neonates and reduces their tolerance to cold temperatures. The basal metabolic rate (rate of energy expenditure per unit time) is higher in smaller animals because they have a higher surface/mass ratio which results in increased corporal heat loss (28–30) in other words, large surface area relative to mass shows a disadvantage in retaining heat (25, 31). Consequently, calves with low body weight may be more susceptible to hypothermia when heat loss exceeds heat production (32).

On the other hand, it is necessary to mention that the surface temperature was significantly lower during days 0, 1, and 2 compared to the rest of the days (*p* < 0.05). This event is probably due to age since the low critical temperature threshold is higher due to the development of thermoregulatory capacity (2). In other words, the growth of the animal leads to an increase in subcutaneous fat reserves, but also an increase in the thickness of the skin and the length of the hair that allow a greater tolerance to cold (27). Johanson and Berger (33) determined that birth weight can be considered a predictor of perinatal mortality, since animals with higher weights have a reduced incidence of mortality due to hypothermia, as has been reported for cattle in eastern Montana. In this study, animals that weighed over 17.42 kg experienced lower temperature decreases when exposed to cold stress (34). Likewise, for Nili-Ravi buffaloes, Hassan et al. (35) indicated that birth weight had a significant effect on mortality rates, as they found a relationship between low birth weight, poor thermoregulation and deficient dam-calf bonding. It has been suggested that newborns with low birth weight and those with insufficient milk supply from the mother are prone to perform compensatory behaviors, such as allosuckling, to prevent nutritional deficiency (36, 37). Similarly, Chnitier et al. (38) found that newborns lambs with low weights had a higher mortality rate (52.2%) than medium- and high-weight animals (24.4 and 1.8%, respectively). Those authors further determined that during winter, when lambs were exposed to a decrease in environmental temperature, the mortality rate was 23.5%, 14% higher than that during the summer.

As our findings show, energy resources differ depending on the weight of newborns, so those with higher weight have a greater chance of using the energy that is available to them to compensate for temperature changes at birth (39). For example, newborn piglets weighing <1 kg have lower energy resources and greater sensitivity to cold stress during the 1st h of life (40). Similarly, data from newborn Holstein-Friesian calves immersed in water at a temperature of 15–17°C to induce cold stress showed 3-fold increases in catecholamine and blood glucose concentrations, suggesting that hypothermia activates the autonomic nervous system to increase blood glucose levels that contribute to shivering and non-shivering thermogenesis (41, 42).

Another factor linked to birth weight that can be altered is the newborn's vitality and ability to reach the mother's udder (43). Ingesting colostrum helps maintain metabolic resources that aid thermoregulation (7). When this behavior is limited, the ability of young animals to acquire more nutrients is reduced (1). A study by Stanko et al. (44) showed that adding exogenous sources of energy in the form of glucose or colostrum maintained thermostability during the 1st day after birth in newborn Brahman calves exposed to a cold environment (5–25°C). A study by Schmidek et al. (45) in cattle identified factors predisposing to the delayed first intake of colostrum and their implications for the calf mortality rate. Bueno et al. (46) also emphasize the importance of first-colostrum feeding latency, and its effect on calf cortisol levels, with adverse effects on immunoglobulin absorption.

In the present study, regardless of birth weight, the calves presented higher than surface temperatures on days 3, 4, and 5, possibly influenced by the quality of nutrition provided during the 1st days of life. Conte et al. (47) reported that the ingestion of good-quality colostrum can mitigate the negative impact of thermal stress because its lipidic and niacin contents participate in the metabolic and vasodilator responses that aid thermoregulation. The energy provided by colostrum is another element that contributes to obtaining resources and maintaining the thermal balance. According to Silva and Bittar (2), a domestic bovine calf requires 4.6–4.7 Mcal/kg to maintain thermoneutrality. In this regard, studies that have focused on colostrum composition have reported fat, protein, and total solids percentages of 11.31, 8.73, and 25.31%, respectively. These three elements also provide neonates to energy (48). These factors could explain the significantly lower temperatures on days 0, 1, and 2, suggesting a compensatory effect of colostrum ingestion that, in a study of newborn Holstein calves, brought about an increase of ~1°C in prescapular temperature (49). The differences found in the days after birth support the hypothesis that colostrum consumption is essential for newborn ruminants to achieve thermoregulatory success (1). Other experimental data have shown that feeding neonates high-fat diets resulted in an increase in body temperature of 0.5°C under exposure to cold climates, an environmental factor also associated with enhanced muscle development in newborns (50, 51).

Therefore, the results presented affirm that the birth weight of buffalo calves is related to the amount of energy available at birth and can affect their thermoregulatory capacity. In addition, said thermoregulatory capacity is developed according to the age of the animals, which can lead to morphophysiological changes.

### 4.2. Temperature variation according to the thermal window

According to Mota-Rojas et al. (52), ruminants have some anatomical regions with a high disposition of blood capillaries, arteriovenous anastomoses, and glabrous skin. These areas are known as thermal windows. These regions facilitate heat exchange between the animal and its environment; therefore, exposure to cold temperatures generates a peripheral vasoconstriction response that decreases blood flow (41).

In the present study, temperatures of the lacrimal gland and lacrimal caruncle did not differ between groups Q_1_ and Q_2_. Moreover, the temperatures recorded in these two thermal windows were higher than those in the other evaluated regions. This suggests that the ocular region could be a useful window for non-invasive assessment of core temperature (23, 53). The difference between these two regions may be attributable to the activation of the sympathetic nervous system (SNS) and the consequent secretion of catecholamines that participate in peripheral vasoconstriction (54–56). This vasomotor response preserves the core temperature in metabolically active organs, such as the brain, heart, and abdomen (28, 57). The effects of this response were described by Shu et al. (58). This body of evidence suggests that the high temperatures exhibited in these two windows are due to their proximity to the brain (59).

Difference between the responses of the lacrimal caruncle and lacrimal gland may also reflect the fact that the latter is irrigated by the infraorbital artery, a structure innervated by the sympathetic branch of the facial nerve (52, 58, 60). Given these anatomical traits, these regions could be helpful for indirectly assessing autonomic nervous system activity as a stress response (61, 62).

The ocular or periocular window can provide a better approximation of core temperature (52, 59). Hoffman et al. (63), for example, established that the IRT readings (mean = 37°C) taken from the eyes of 22 cows and nine calves demonstrated that this is a suitable area for monitoring body temperature, unlike the ear and shoulder. Recently, studies have been done in buffaloes, in which it has been determined that the orbital thermal window could be a validated zone in this specie to record temperature (14, 52, 59).

In contrast to these regions, the auditory canal and pelvic limb registered low temperatures. The recorded values indicate heat loss through the skin due to dermal vasoconstriction and closing of arteriovenous anastomoses (28). IRT readings from the ears and limbs of newborn piglets should be useful to recognize the early stages of hypothermia, although it is important to mention that the concave shape of the auditory canal could alter radiation in that zone (23). For this reason, evaluating the thermal response of the limbs, where blood flow may be reduced due to the vasomotor changes that occur under exposure to cold, could be an alternative (1), even though this possibility requires further study.

The nostrils showed significantly lower temperatures than those recorded in the ocular, lacrimal caruncle, and auricular regions. These results are attributed to the tachypnea response as a means of achieving thermoneutrality (64). In this regard, the increase in oxygen intake compensates for one of the main consequences of hypothermia; namely, hypoxemia (57, 65). Even under conditions of regular respiratory rate, the convective processes associated with the inhaled and exhaled airflows, provide evaporation of fluid present in the nostrils that cause the corresponding cooling. The response in this window reflects increased airflow, which could be helpful in monitoring the breathing patterns of animals (66). On this topic, Kim and Hidaka (67) used IRT and computer vision systems to state breathing patterns in cattle. The authors obtained an accuracy of 76%. However, when evaluating temperatures in this region, elements such as the moist surface must be considered (68), as they can alter measurements due to heat dissipation by evaporation (69).

The IRT technique is useful because it responds to the vascular structure of each thermal window, thus allowing for more reliable approximations to detect changes in the body temperature of newborn buffaloes associated with hypothermic states.

### 4.3. Effect of time of day on thermoregulation in newborn buffaloes

The thermal responses of the six windows evaluated were affected by the time of the day (morning *vs*. afternoon). In addition to this, it was possible to observe that the temperatures of all the thermal windows presented positive correlation with the time of day (*p* < 0.05). The temperature readings of the buffalo calves were significantly higher in the afternoon. A possible explanation for this is the effect of environmental factors. Villanueva-García et al. (23), Mota-Rojas et al. (69), and Kozat (15) mentioned that elements such as wind and rain can cause hypothermia in newborns, whereas solar radiation has the opposite effect (70).

In the six thermal windows, during the afternoon, heat loss by evaporation is attenuated, so the amount of blood in the dermal tissue increases due to the activation of the SNA (71, 72). A study of Murrah water buffaloes reported that exposure to 45°C during the summer increased blood flow by 5–17 units (in the dorsal, abdominal, and auricular areas), as well as the animals' respiratory frequency (73). Marai and Heeeb (74) mention that the characteristics for the water buffalo to remain in an optimal productive state are an air temperature of 13–18°C, relative humidity of 55–60%, and an air speed of 5–8 km/h. An environmental temperature lower than 15°C is a crucial factor that could challenge thermoregulatory mechanisms in newborn, while wind speed and solar radiation could affect the IRT reading. This could be a limitation within the present study and an element to consider for future studies.

The ability of water buffaloes to maintain thermoneutrality during periods of intense heat also depends on characteristics such as the presence of a thick epidermis, low hair density, and abundant melanin (14, 52). Interestingly, these features may help in a certain way to increase resilience to cold because the difficulty that these animals experience in dissipating heat could be beneficial by preventing greater heat loss through evaporation (73).

According to our findings, the time of the day influences the thermostability of newborn water buffaloes (75); therefore, this must be considered an important factor for the perinatal management of the offspring of this species.

## 5. Conclusion

According to the results obtained, the birth weight of water buffalo calves is a factor that can alter their thermoregulation, as calves with higher weight at birth achieve thermostability more quickly than those with lower weights. However, individual characteristics are not the only factor that should be considered, as the half-period of day can also alter the surface temperatures of animals, since they tend to be lower in the morning and higher in the afternoon. Considering these findings, IRT can be used as an additional tool to monitor normothermic states in newborn water buffaloes, where timely diagnosis of critical states can promote better productive management of buffalo calves in the face of possible exposed to cold. The findings in the present article show that lacrimal caruncle and periocular surface can be considered as more stable thermal windows to monitor the thermal state of the newborns, followed by nostrils.

## Data availability statement

The original contributions presented in the study are included in the article/supplementary material, further inquiries can be directed to the corresponding author.

## Ethics statement

The animals monitored in this study were not touched or stressed, as infrared thermography is a non-invasive technique; therefore, the protocol did not require approval from an Ethics Committee. During the study, handling of the animals was carried out under the guidelines of the Official Mexican Standard, NOM062-ZOO-1999, which stipulates the technical specifications for the production, care, and ethical use of animals.

## Author contributions

All authors contributed to the conceptualization, writing, reading, and approval of the final manuscript.
